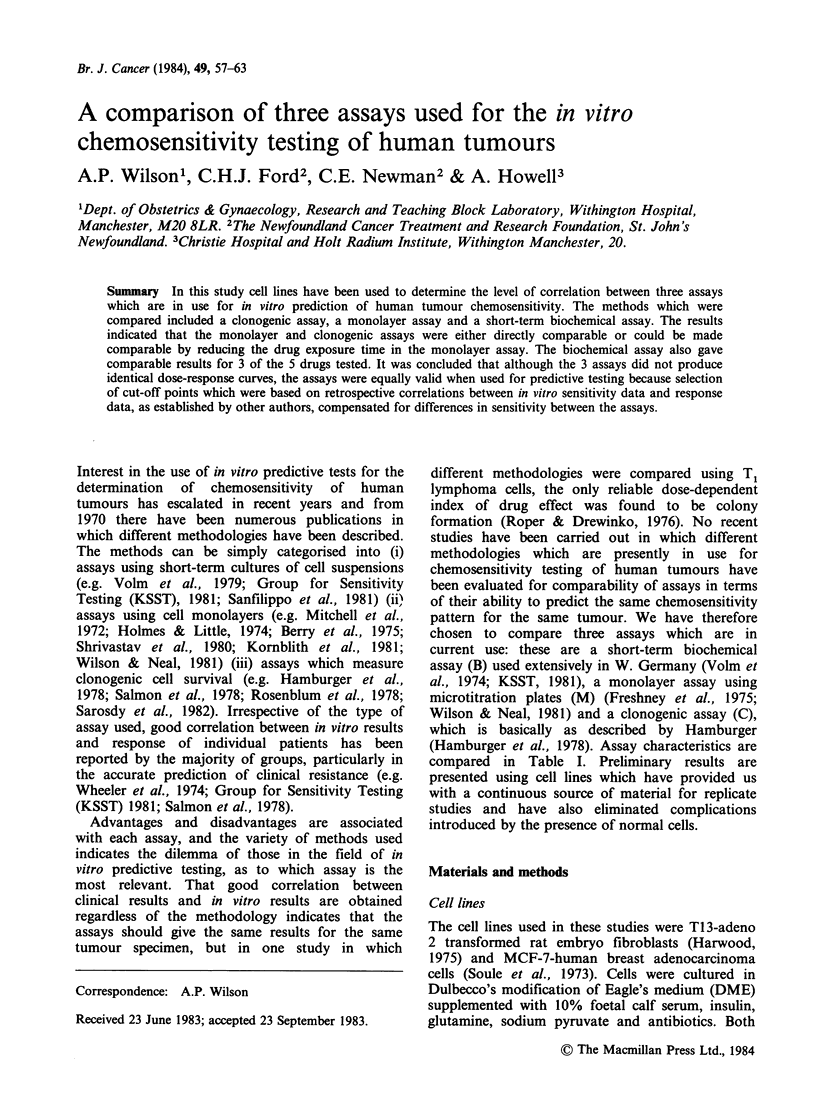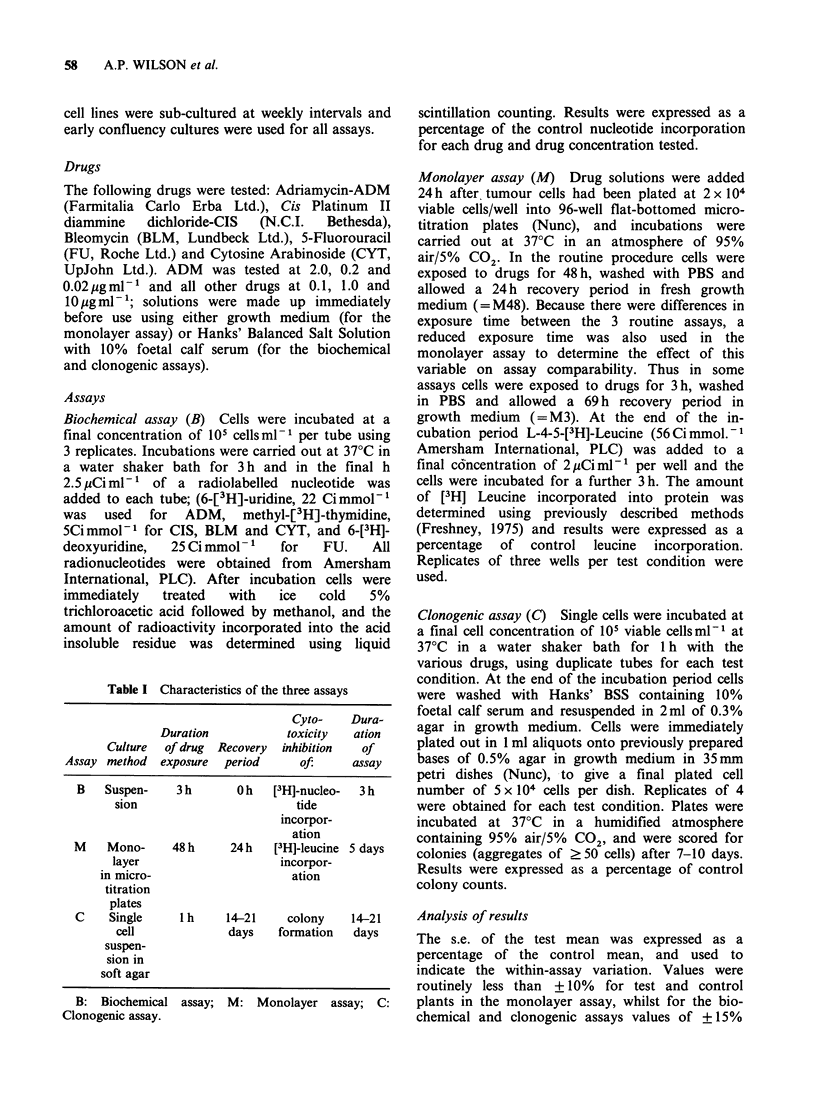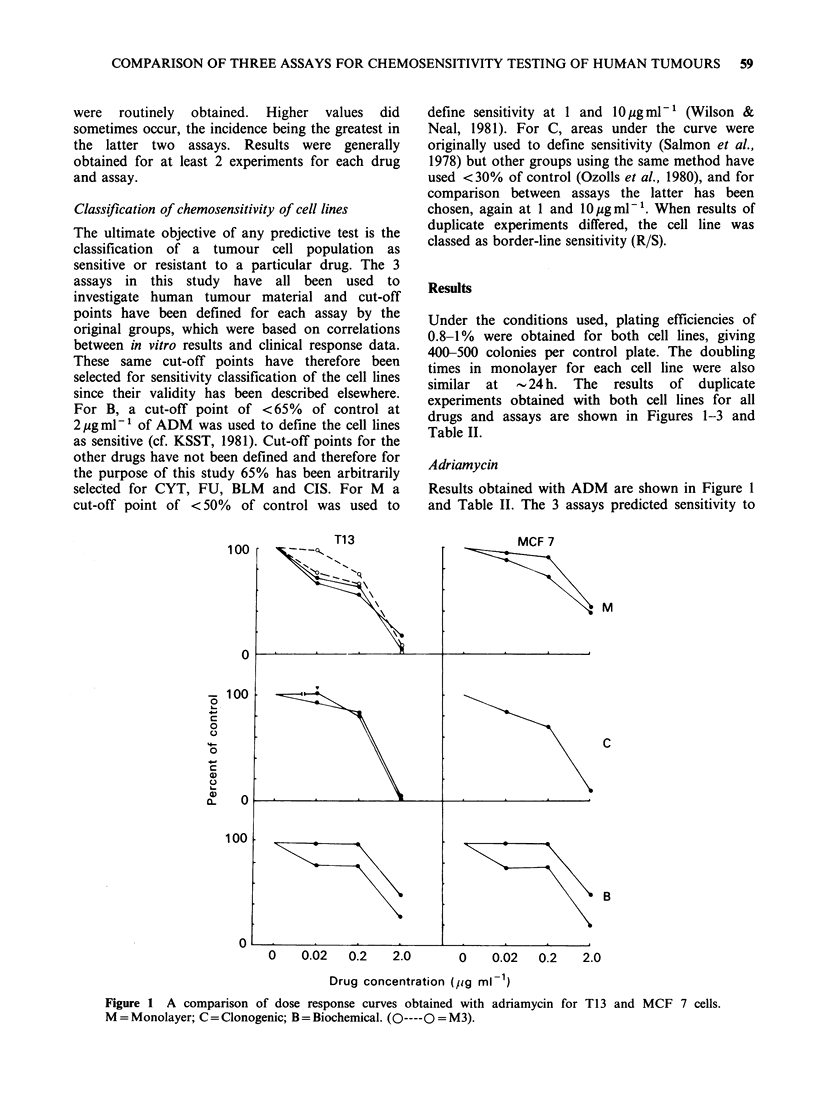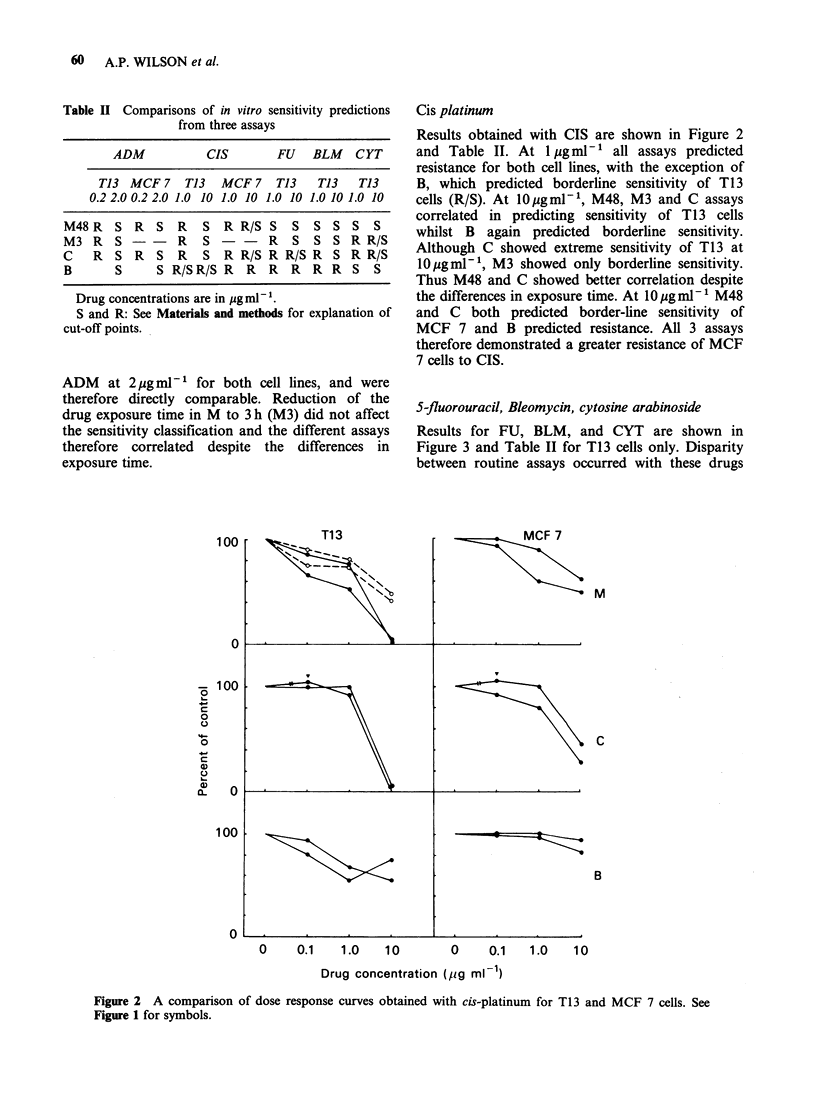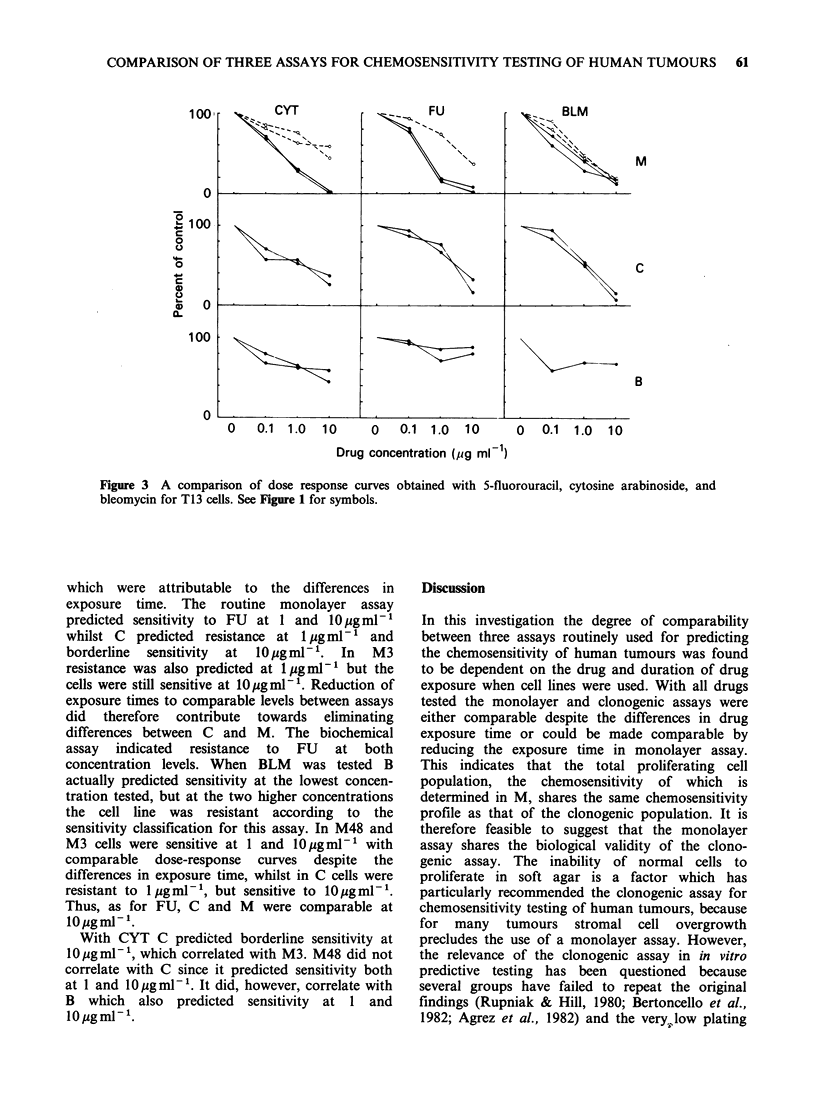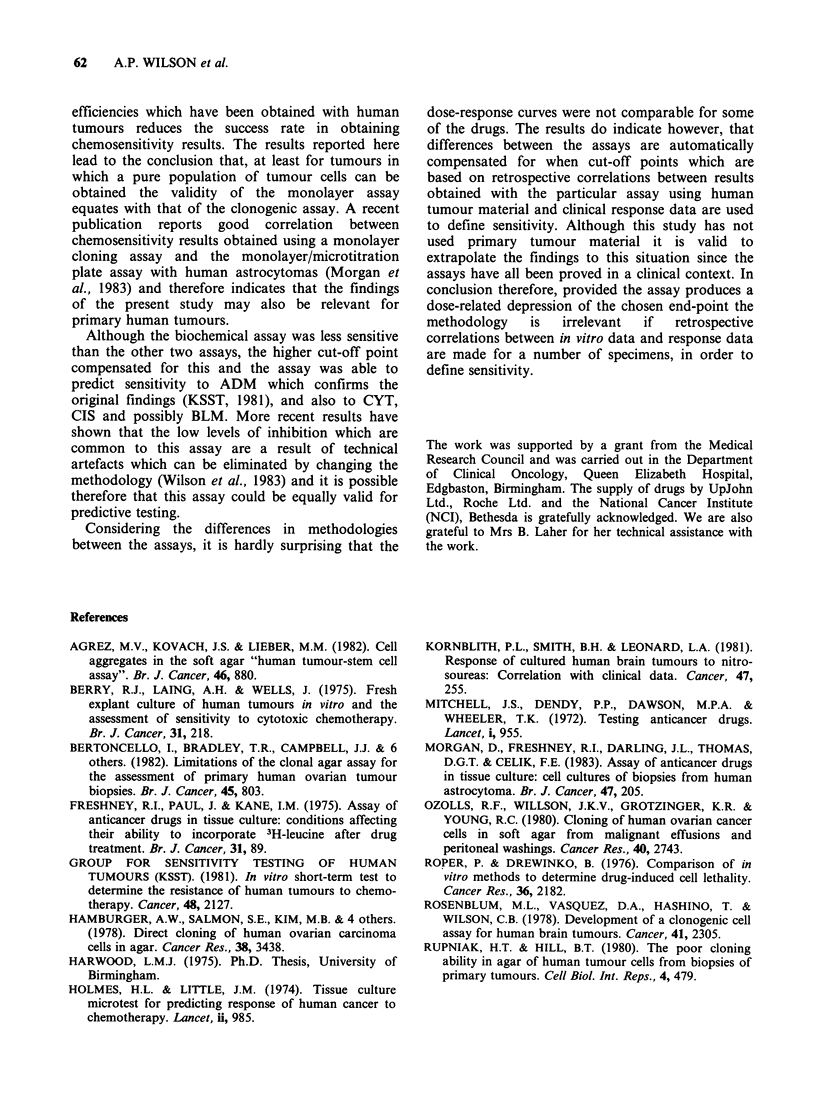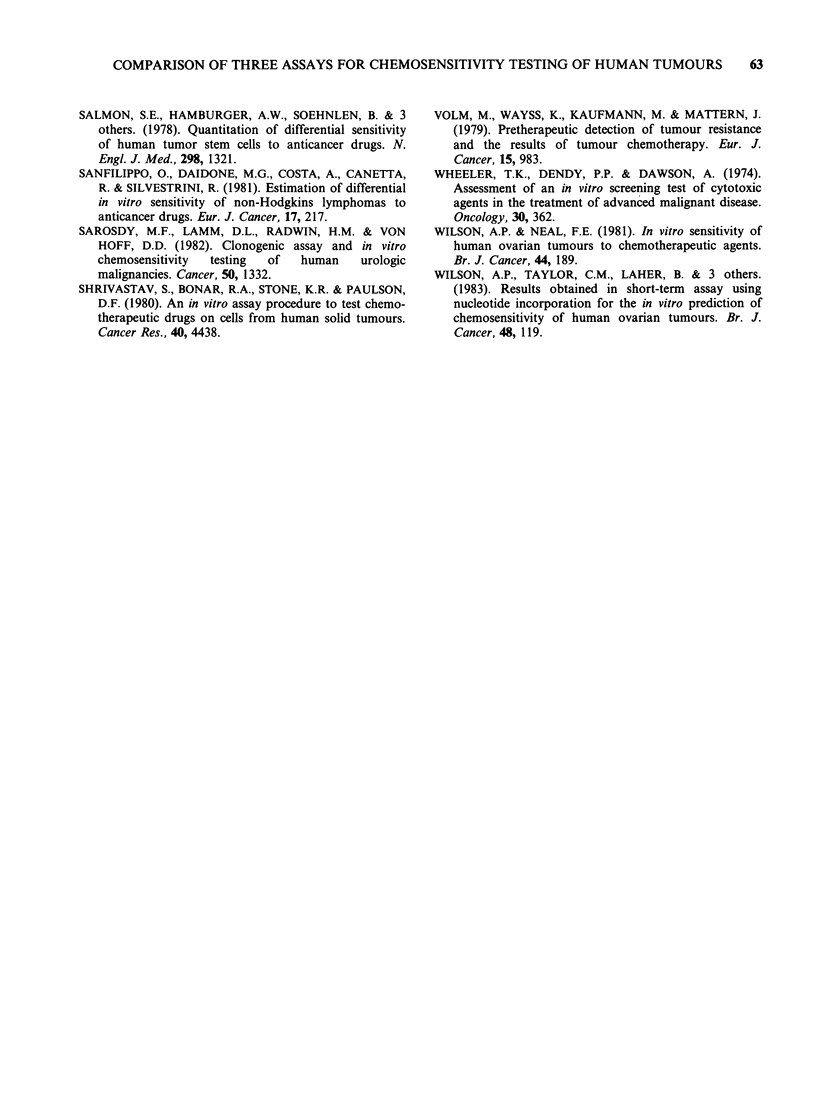# A comparison of three assays used for the in vitro chemosensitivity testing of human tumours.

**DOI:** 10.1038/bjc.1984.9

**Published:** 1984-01

**Authors:** A. P. Wilson, C. H. Ford, C. E. Newman, A. Howell

## Abstract

In this study cell lines have been used to determine the level of correlation between three assays which are in use for in vitro prediction of human tumour chemosensitivity. The methods which were compared included a clonogenic assay, a monolayer assay and a short-term biochemical assay. The results indicated that the monolayer and clonogenic assays were either directly comparable or could be made comparable by reducing the drug exposure time in the monolayer assay. The biochemical assay also gave comparable results for 3 of the 5 drugs tested. It was concluded that although the 3 assays did not produce identical dose-response curves, the assays were equally valid when used for predictive testing because selection of cut-off points which were based on retrospective correlations between in vitro sensitivity data and response data, as established by other authors, compensated for differences in sensitivity between the assays.


*Br. J. Cancer* (1984), **49**, 57–63

### A comparison of three assays used for the *in vitro* chemosensitivity testing of human tumours

A.P. Wilson[1], C.H.J. Ford[2], C.E. Newman[2] & A. Howell[3]

[1]*Dept. of Obstetrics & Gynaecology, Research and Teaching Block Laboratory, Withington Hospital, Manchester, M20 8LR.* [2]*The Newfoundland Cancer Treatment and Research Foundation, St. John's Newfoundland.* [3]*Christie Hospital and Holt Radium Institute, Withington Manchester, 20.*

**Summary** In this study cell lines have been used to determine the level of correlation between three assays which are in use for *in vitro* prediction of human tumour chemosensitivity. The methods which were compared included a clonogenic assay, a monolayer assay and a short-term biochemical assay. The results indicated that the monolayer and clonogenic assays were either directly comparable or could be made comparable by reducing the drug exposure time in the monolayer assay. The biochemical assay also gave comparable results for 3 of the 5 drugs tested. It was concluded that although the 3 assays did not produce identical dose-response curves, the assays were equally valid when used for predictive testing because selection of cut-off points which were based on retrospective correlations between *in vitro* sensitivity data and response data, as established by other authors, compensated for differences in sensitivity between the assays.

Interest in the use of *in vitro* predictive tests for the determination of chemosensitivity of human tumours has escalated in recent years and from 1970 there have been numerous publications in which different methodologies have been described. The methods can be simply categorised into (i) assays using short-term cultures of cell suspensions (e.g. Volm *et al.*, 1979; Group for Sensitivity Testing (KSST), 1981; Sanfilippo *et al.*, 1981) (ii) assays using cell monolayers (e.g. Mitchell *et al.*, 1972; Holmes & Little, 1974; Berry *et al.*, 1975; Shrivastav *et al.*, 1980; Kornblith *et al.*, 1981; Wilson & Neal, 1981) (iii) assays which measure clonogenic cell survival (e.g. Hamburger *et al.*, 1978; Salmon *et al.*, 1978; Rosenblum *et al.*, 1978; Sarosdy *et al.*, 1982). Irrespective of the type of assay used, good correlation between *in vitro* results and response of individual patients has been reported by the majority of groups, particularly in the accurate prediction of clinical resistance (e.g. Wheeler *et al.*, 1974; Group for Sensitivity Testing (KSST) 1981; Salmon *et al.*, 1978).

Advantages and disadvantages are associated with each assay, and the variety of methods used indicates the dilemma of those in the field of *in vitro* predictive testing, as to which assay is the most relevant. That good correlation between clinical results and *in vitro* results are obtained regardless of the methodology indicates that the assays should give the same results for the same tumour specimen, but in one study in which different methodologies were compared using $T_1$ lymphoma cells, the only reliable dose-dependent index of drug effect was found to be colony formation (Roper & Drewinko, 1976). No recent studies have been carried out in which different methodologies which are presently in use for chemosensitivity testing of human tumours have been evaluated for comparability of assays in terms of their ability to predict the same chemosensitivity pattern for the same tumour. We have therefore chosen to compare three assays which are in current use: these are a short-term biochemical assay (B) used extensively in W. Germany (Volm *et al.*, 1974; KSST, 1981), a monolayer assay using microtitration plates (M) (Freshney *et al.*, 1975; Wilson & Neal, 1981) and a clonogenic assay (C), which is basically as described by Hamburger (Hamburger *et al.*, 1978). Assay characteristics are compared in Table I. Preliminary results are presented using cell lines which have provided us with a continuous source of material for replicate studies and have also eliminated complications introduced by the presence of normal cells.

### Materials and methods

### Cell lines

The cell lines used in these studies were T13-adeno 2 transformed rat embryo fibroblasts (Harwood, 1975) and MCF-7-human breast adenocarcinoma cells (Soule *et al.*, 1973). Cells were cultured in Dulbecco's modification of Eagle's medium (DME) supplemented with 10% foetal calf serum, insulin, glutamine, sodium pyruvate and antibiotics. Both

Correspondence: A.P. Wilson

Received 23 June 1983; accepted 23 September 1983.

© The Macmillan Press Ltd., 1984

cell lines were sub-cultured at weekly intervals and early confluency cultures were used for all assays.

*Drugs*

The following drugs were tested: Adriamycin-ADM (Farmitalia Carlo Erba Ltd.), *Cis* Platinum II diammine dichloride-CIS (N.C.I. Bethesda), Bleomycin (BLM, Lundbeck Ltd.), 5-Fluorouracil (FU, Roche Ltd.) and Cytosine Arabinoside (CYT, UpJohn Ltd.). ADM was tested at 2.0, 0.2 and 0.02 $\mu g\,ml^{-1}$ and all other drugs at 0.1, 1.0 and 10 $\mu g\,ml^{-1}$; solutions were made up immediately before use using either growth medium (for the monolayer assay) or Hanks' Balanced Salt Solution with 10% foetal calf serum (for the biochemical and clonogenic assays).

*Assays*

*Biochemical assay* (*B*) Cells were incubated at a final concentration of $10^5$ cells $ml^{-1}$ per tube using 3 replicates. Incubations were carried out at 37°C in a water shaker bath for 3 h and in the final h 2.5 $\mu Ci\,ml^{-1}$ of a radiolabelled nucleotide was added to each tube; (6-[$^3$H]-uridine, 22 Ci $mmol^{-1}$ was used for ADM, methyl-[$^3$H]-thymidine, 5Ci $mmol^{-1}$ for CIS, BLM and CYT, and 6-[$^3$H]-deoxyuridine, 25 Ci $mmol^{-1}$ for FU. All radionucleotides were obtained from Amersham International, PLC). After incubation cells were immediately treated with ice cold 5% trichloroacetic acid followed by methanol, and the amount of radioactivity incorporated into the acid insoluble residue was determined using liquid scintillation counting. Results were expressed as a percentage of the control nucleotide incorporation for each drug and drug concentration tested.

*Monolayer assay* (*M*) Drug solutions were added 24 h after tumour cells had been plated at $2 \times 10^4$ viable cells/well into 96-well flat-bottomed microtitration plates (Nunc), and incubations were carried out at 37°C in an atmosphere of 95% air/5% $CO_2$. In the routine procedure cells were exposed to drugs for 48 h, washed with PBS and allowed a 24 h recovery period in fresh growth medium (=M48). Because there were differences in exposure time between the 3 routine assays, a reduced exposure time was also used in the monolayer assay to determine the effect of this variable on assay comparability. Thus in some assays cells were exposed to drugs for 3 h, washed in PBS and allowed a 69 h recovery period in growth medium (=M3). At the end of the incubation period L-4-5-[$^3$H]-Leucine (56 Ci $mmol.^{-1}$ Amersham International, PLC) was added to a final concentration of 2 $\mu Ci\,ml^{-1}$ per well and the cells were incubated for a further 3 h. The amount of [$^3$H] Leucine incorporated into protein was determined using previously described methods (Freshney, 1975) and results were expressed as a percentage of control leucine incorporation. Replicates of three wells per test condition were used.

*Clonogenic assay* (*C*) Single cells were incubated at a final cell concentration of $10^5$ viable cells $ml^{-1}$ at 37°C in a water shaker bath for 1 h with the various drugs, using duplicate tubes for each test condition. At the end of the incubation period cells were washed with Hanks' BSS containing 10% foetal calf serum and resuspended in 2 ml of 0.3% agar in growth medium. Cells were immediately plated out in 1 ml aliquots onto previously prepared bases of 0.5% agar in growth medium in 35 mm petri dishes (Nunc), to give a final plated cell number of $5 \times 10^4$ cells per dish. Replicates of 4 were obtained for each test condition. Plates were incubated at 37°C in a humidified atmosphere containing 95% air/5% $CO_2$, and were scored for colonies (aggregates of $\geq 50$ cells) after 7–10 days. Results were expressed as a percentage of control colony counts.

**Table I** Characteristics of the three assays

| *Assay* | *Culture method* | *Duration of drug exposure* | *Recovery period* | *Cytotoxicity inhibition of:* | *Duration of assay* |
|---|---|---|---|---|---|
| B | Suspension | 3 h | 0 h | [$^3$H]-nucleotide incorporation | 3 h |
| M | Monolayer in microtitration plates | 48 h | 24 h | [$^3$H]-leucine incorporation | 5 days |
| C | Single cell suspension in soft agar | 1 h | 14–21 days | colony formation | 14–21 days |

B: Biochemical assay; M: Monolayer assay; C: Clonogenic assay.

*Analysis of results*

The s.e. of the test mean was expressed as a percentage of the control mean, and used to indicate the within-assay variation. Values were routinely less than ±10% for test and control plants in the monolayer assay, whilst for the biochemical and clonogenic assays values of ±15%

were routinely obtained. Higher values did sometimes occur, the incidence being the greatest in the latter two assays. Results were generally obtained for at least 2 experiments for each drug and assay.

*Classification of chemosensitivity of cell lines*

The ultimate objective of any predictive test is the classification of a tumour cell population as sensitive or resistant to a particular drug. The 3 assays in this study have all been used to investigate human tumour material and cut-off points have been defined for each assay by the original groups, which were based on correlations between *in vitro* results and clinical response data. These same cut-off points have therefore been selected for sensitivity classification of the cell lines since their validity has been described elsewhere. For B, a cut-off point of <65% of control at $2\,\mu g\,ml^{-1}$ of ADM was used to define the cell lines as sensitive (cf. KSST, 1981). Cut-off points for the other drugs have not been defined and therefore for the purpose of this study 65% has been arbitrarily selected for CYT, FU, BLM and CIS. For M a cut-off point of <50% of control was used to define sensitivity at 1 and $10\,\mu g\,ml^{-1}$ (Wilson & Neal, 1981). For C, areas under the curve were originally used to define sensitivity (Salmon *et al.*, 1978) but other groups using the same method have used <30% of control (Ozolls *et al.*, 1980), and for comparison between assays the latter has been chosen, again at 1 and $10\,\mu g\,ml^{-1}$. When results of duplicate experiments differed, the cell line was classed as border-line sensitivity (R/S).

### Results

Under the conditions used, plating efficiencies of 0.8–1% were obtained for both cell lines, giving 400–500 colonies per control plate. The doubling times in monolayer for each cell line were also similar at ~24 h. The results of duplicate experiments obtained with both cell lines for all drugs and assays are shown in Figures 1–3 and Table II.

*Adriamycin*

Results obtained with ADM are shown in Figure 1 and Table II. The 3 assays predicted sensitivity to

**Figure 1** A comparison of dose response curves obtained with adriamycin for T13 and MCF 7 cells. M = Monolayer; C = Clonogenic; B = Biochemical. (O----O = M3).

**Table II** Comparisons of *in vitro* sensitivity predictions from three assays

| | *ADM* | | | | *CIS* | | | | *FU* | | *BLM* | | *CYT* | |
|---|---|---|---|---|---|---|---|---|---|---|---|---|---|---|
| | *T13* | | *MCF 7* | | *T13* | | *MCF 7* | | *T13* | | *T13* | | *T13* | |
| | *0.2* | *2.0* | *0.2* | *2.0* | *1.0* | *10* | *1.0* | *10* | *1.0* | *10* | *1.0* | *10* | *1.0* | *10* |
| M48 | R | S | R | S | R | S | R | R/S | S | S | S | S | S | S |
| M3 | R | S | — | — | R | S | — | — | R | S | S | S | R | R/S |
| C | R | S | R | S | R | S | R | R/S | R | R/S | R | S | R | R/S |
| B | | S | | S | R/S | R/S | R | R | R | R | R | R | S | S |

Drug concentrations are in $\mu g\,ml^{-1}$.

S and R: See **Materials and methods** for explanation of cut-off points.

ADM at $2\,\mu g\,ml^{-1}$ for both cell lines, and were therefore directly comparable. Reduction of the drug exposure time in M to 3 h (M3) did not affect the sensitivity classification and the different assays therefore correlated despite the differences in exposure time.

### Cis *platinum*

Results obtained with CIS are shown in Figure 2 and Table II. At $1\,\mu g\,ml^{-1}$ all assays predicted resistance for both cell lines, with the exception of B, which predicted borderline sensitivity of T13 cells (R/S). At $10\,\mu g\,ml^{-1}$, M48, M3 and C assays correlated in predicting sensitivity of T13 cells whilst B again predicted borderline sensitivity. Although C showed extreme sensitivity of T13 at $10\,\mu g\,ml^{-1}$, M3 showed only borderline sensitivity. Thus M48 and C showed better correlation despite the differences in exposure time. At $10\,\mu g\,ml^{-1}$ M48 and C both predicted border-line sensitivity of MCF 7 and B predicted resistance. All 3 assays therefore demonstrated a greater resistance of MCF 7 cells to CIS.

### *5-fluorouracil, Bleomycin, cytosine arabinoside*

Results for FU, BLM, and CYT are shown in Figure 3 and Table II for T13 cells only. Disparity between routine assays occurred with these drugs

**Figure 2** A comparison of dose response curves obtained with *cis*-platinum for T13 and MCF 7 cells. See **Figure 1** for symbols.

**Figure 3** A comparison of dose response curves obtained with 5-fluorouracil, cytosine arabinoside, and bleomycin for T13 cells. See **Figure 1** for symbols.

which were attributable to the differences in exposure time. The routine monolayer assay predicted sensitivity to FU at 1 and $10\,\mu g\,ml^{-1}$ whilst C predicted resistance at $1\,\mu g\,ml^{-1}$ and borderline sensitivity at $10\,\mu g\,ml^{-1}$. In M3 resistance was also predicted at $1\,\mu g\,ml^{-1}$ but the cells were still sensitive at $10\,\mu g\,ml^{-1}$. Reduction of exposure times to comparable levels between assays did therefore contribute towards eliminating differences between C and M. The biochemical assay indicated resistance to FU at both concentration levels. When BLM was tested B actually predicted sensitivity at the lowest concentration tested, but at the two higher concentrations the cell line was resistant according to the sensitivity classification for this assay. In M48 and M3 cells were sensitive at 1 and $10\,\mu g\,ml^{-1}$ with comparable dose-response curves despite the differences in exposure time, whilst in C cells were resistant to $1\,\mu g\,ml^{-1}$, but sensitive to $10\,\mu g\,ml^{-1}$. Thus, as for FU, C and M were comparable at $10\,\mu g\,ml^{-1}$.

With CYT C predicted borderline sensitivity at $10\,\mu g\,ml^{-1}$, which correlated with M3. M48 did not correlate with C since it predicted sensitivity both at 1 and $10\,\mu g\,ml^{-1}$. It did, however, correlate with B which also predicted sensitivity at 1 and $10\,\mu g\,ml^{-1}$.

### Discussion

In this investigation the degree of comparability between three assays routinely used for predicting the chemosensitivity of human tumours was found to be dependent on the drug and duration of drug exposure when cell lines were used. With all drugs tested the monolayer and clonogenic assays were either comparable despite the differences in drug exposure time or could be made comparable by reducing the exposure time in monolayer assay. This indicates that the total proliferating cell population, the chemosensitivity of which is determined in M, shares the same chemosensitivity profile as that of the clonogenic population. It is therefore feasible to suggest that the monolayer assay shares the biological validity of the clonogenic assay. The inability of normal cells to proliferate in soft agar is a factor which has particularly recommended the clonogenic assay for chemosensitivity testing of human tumours, because for many tumours stromal cell overgrowth precludes the use of a monolayer assay. However, the relevance of the clonogenic assay in *in vitro* predictive testing has been questioned because several groups have failed to repeat the original findings (Rupniak & Hill, 1980; Bertoncello *et al.*, 1982; Agrez *et al.*, 1982) and the very low plating efficiencies which have been obtained with human tumours reduces the success rate in obtaining chemosensitivity results. The results reported here lead to the conclusion that, at least for tumours in which a pure population of tumour cells can be obtained the validity of the monolayer assay equates with that of the clonogenic assay. A recent publication reports good correlation between chemosensitivity results obtained using a monolayer cloning assay and the monolayer/microtitration plate assay with human astrocytomas (Morgan *et al.*, 1983) and therefore indicates that the findings of the present study may also be relevant for primary human tumours.

Although the biochemical assay was less sensitive than the other two assays, the higher cut-off point compensated for this and the assay was able to predict sensitivity to ADM which confirms the original findings (KSST, 1981), and also to CYT, CIS and possibly BLM. More recent results have shown that the low levels of inhibition which are common to this assay are a result of technical artefacts which can be eliminated by changing the methodology (Wilson *et al.*, 1983) and it is possible therefore that this assay could be equally valid for predictive testing.

Considering the differences in methodologies between the assays, it is hardly surprising that the dose-response curves were not comparable for some of the drugs. The results do indicate however, that differences between the assays are automatically compensated for when cut-off points which are based on retrospective correlations between results obtained with the particular assay using human tumour material and clinical response data are used to define sensitivity. Although this study has not used primary tumour material it is valid to extrapolate the findings to this situation since the assays have all been proved in a clinical context. In conclusion therefore, provided the assay produces a dose-related depression of the chosen end-point the methodology is irrelevant if retrospective correlations between *in vitro* data and response data are made for a number of specimens, in order to define sensitivity.

The work was supported by a grant from the Medical Research Council and was carried out in the Department of Clinical Oncology, Queen Elizabeth Hospital, Edgbaston, Birmingham. The supply of drugs by UpJohn Ltd., Roche Ltd. and the National Cancer Institute (NCI), Bethesda is gratefully acknowledged. We are also grateful to Mrs B. Laher for her technical assistance with the work.

### References

AGREZ, M.V., KOVACH, J.S. & LIEBER, M.M. (1982). Cell aggregates in the soft agar "human tumour-stem cell assay". *Br. J. Cancer*, **46**, 880.

BERRY, R.J., LAING, A.H. & WELLS, J. (1975). Fresh explant culture of human tumours *in vitro* and the assessment of sensitivity to cytotoxic chemotherapy. *Br. J. Cancer*, **31**, 218.

BERTONCELLO, I., BRADLEY, T.R., CAMPBELL, J.J. & 6 others. (1982). Limitations of the clonal agar assay for the assessment of primary human ovarian tumour biopsies. *Br. J. Cancer*, **45**, 803.

FRESHNEY, R.I., PAUL, J. & KANE, I.M. (1975). Assay of anticancer drugs in tissue culture: conditions affecting their ability to incorporate $^{3}$H-leucine after drug treatment. *Br. J. Cancer*, **31**, 89.

GROUP FOR SENSITIVITY TESTING OF HUMAN TUMOURS (KSST). (1981). *In vitro* short-term test to determine the resistance of human tumours to chemotherapy. *Cancer*, **48**, 2127.

HAMBURGER, A.W., SALMON, S.E., KIM, M.B. & 4 others. (1978). Direct cloning of human ovarian carcinoma cells in agar. *Cancer Res.*, **38**, 3438.

HARWOOD, L.M.J. (1975). Ph.D. Thesis, University of Birmingham.

HOLMES, H.L. & LITTLE, J.M. (1974). Tissue culture microtest for predicting response of human cancer to chemotherapy. *Lancet*, **ii**, 985.

KORNBLITH, P.L., SMITH, B.H. & LEONARD, L.A. (1981). Response of cultured human brain tumours to nitrosoureas: Correlation with clinical data. *Cancer*, **47**, 255.

MITCHELL, J.S., DENDY, P.P., DAWSON, M.P.A. & WHEELER, T.K. (1972). Testing anticancer drugs. *Lancet*, **i**, 955.

MORGAN, D., FRESHNEY, R.I., DARLING, J.L., THOMAS, D.G.T. & CELIK, F.E. (1983). Assay of anticancer drugs in tissue culture: cell cultures of biopsies from human astrocytoma. *Br. J. Cancer*, **47**, 205.

OZOLLS, R.F., WILLSON, J.K.V., GROTZINGER, K.R. & YOUNG, R.C. (1980). Cloning of human ovarian cancer cells in soft agar from malignant effusions and peritoneal washings. *Cancer Res.*, **40**, 2743.

ROPER, P. & DREWINKO, B. (1976). Comparison of *in vitro* methods to determine drug-induced cell lethality. *Cancer Res.*, **36**, 2182.

ROSENBLUM, M.L., VASQUEZ, D.A., HASHINO, T. & WILSON, C.B. (1978). Development of a clonogenic cell assay for human brain tumours. *Cancer*, **41**, 2305.

RUPNIAK, H.T. & HILL, B.T. (1980). The poor cloning ability in agar of human tumour cells from biopsies of primary tumours. *Cell Biol. Int. Reps.*, **4**, 479.

SALMON, S.E., HAMBURGER, A.W., SOEHNLEN, B. & 3 others. (1978). Quantitation of differential sensitivity of human tumor stem cells to anticancer drugs. *N. Engl. J. Med.*, **298**, 1321.

SANFILIPPO, O., DAIDONE, M.G., COSTA, A., CANETTA, R. & SILVESTRINI, R. (1981). Estimation of differential *in vitro* sensitivity of non-Hodgkins lymphomas to anticancer drugs. *Eur. J. Cancer*, **17**, 217.

SAROSDY, M.F., LAMM, D.L., RADWIN, H.M. & VON HOFF, D.D. (1982). Clonogenic assay and *in vitro* chemosensitivity testing of human urologic malignancies. *Cancer*, **50**, 1332.

SHRIVASTAV, S., BONAR, R.A., STONE, K.R. & PAULSON, D.F. (1980). An *in vitro* assay procedure to test chemotherapeutic drugs on cells from human solid tumours. *Cancer Res.*, **40**, 4438.

VOLM, M., WAYSS, K., KAUFMANN, M. & MATTERN, J. (1979). Pretherapeutic detection of tumour resistance and the results of tumour chemotherapy. *Eur. J. Cancer*, **15**, 983.

WHEELER, T.K., DENDY, P.P. & DAWSON, A. (1974). Assessment of an *in vitro* screening test of cytotoxic agents in the treatment of advanced malignant disease. *Oncology*, **30**, 362.

WILSON, A.P. & NEAL, F.E. (1981). *In vitro* sensitivity of human ovarian tumours to chemotherapeutic agents. *Br. J. Cancer*, **44**, 189.

WILSON, A.P., TAYLOR, C.M., LAHER, B. & 3 others. (1983). Results obtained in short-term assay using nucleotide incorporation for the *in vitro* prediction of chemosensitivity of human ovarian tumours. *Br. J. Cancer*, **48**, 119.